# Multiple sclerosis as a biological and clinical continuum: from risk factors to the early stages of disease

**DOI:** 10.3389/fneur.2026.1812340

**Published:** 2026-05-28

**Authors:** Ana Belén Caminero, María Luisa Martínez Ginés, Montserrat Gómez Gutiérrez, Inmaculada García Castañón

**Affiliations:** 1 Multiple Sclerosis Unit, Department of Neurology, Complejo Asistencial de Ávila, Ávila, Spain; 2 Multiple Sclerosis Unit, Department of Neurology, Hospital General Universitario Gregorio Marañón, Madrid, Spain; 3 Department of Neurology, Hospital San Pedro de Alcántara, Cáceres, Spain; 4 Department of Neurology, Hospital Universitario de Fuenlabrada, Madrid, Spain

**Keywords:** biomarkers, clinically isolated syndrome, environment, genetics, multiple sclerosis, neuroimaging, prodrome, radiologically isolated syndrome

## Abstract

Multiple sclerosis (MS) has traditionally been diagnosed after the onset of clinical symptoms, supported by characteristic radiological findings. However, mounting evidence suggests that MS-related biological processes may precede the first overt neurological manifestations by several years. The disease appears to unfold along a biological continuum that includes genetic susceptibility, environmental exposures, and possible early manifestations characterized by subtle, nonspecific symptoms that precede detectable abnormalities such as MRI lesions or early biomarker changes. This review summarizes current knowledge about the temporal evolution of MS, from early risk factors through the radiologically isolated syndrome (RIS) and clinically isolated syndrome (CIS), to the onset of clinically definite MS. We discuss emerging biomarkers, advances in neuroimaging, and evolving insights into early immunopathological mechanisms. Conceptualizing MS as a continuum may facilitate improved risk stratification and the development of targeted monitoring strategies in individuals at higher risk. Nevertheless, this model remains limited by the absence of biomarkers with sufficient specificity to reliably characterize preclinical or prodromal biological disease activity, and of validated tools to translate such findings into clinical practice.

## 1 Introduction/conceptual framework

Multiple sclerosis (MS) is a chronic immune-mediated demyelinating disease of the central nervous system (CNS). Although MS primarily affects young adults, it can also manifest in pediatric patients and in older individuals (after age 50, referred to as late-onset MS).

MS has traditionally been defined by clinical relapses or progressive neurological decline following the appearance of overt symptoms. However, growing evidence supports the concept that MS unfolds along a continuum that extends beyond the first clinical manifestation: from a genetically and environmentally determined predisposition, through a prodromal phase characterized by subtle or nonspecific symptoms, to a subsequent subclinical stage detectable by emerging biomarkers, most notably magnetic resonance imaging (MRI) lesions characteristic of radiologically isolated syndrome (RIS). Ultimately, the first clinical relapse, or clinically isolated syndrome (CIS) marks the transition to what is classically recognized as MS.

Recent updates to diagnostic criteria further support this perspective, acknowledging that, in specific circumstances, asymptomatic individuals with characteristic radiological and cerebrospinal fluid (CSF) findings may fulfil MS diagnostic criteria (1), reflecting underlying biological disease activity; however, the interpretation and clinical implications of such findings remain under debate.

**Figure 1:**
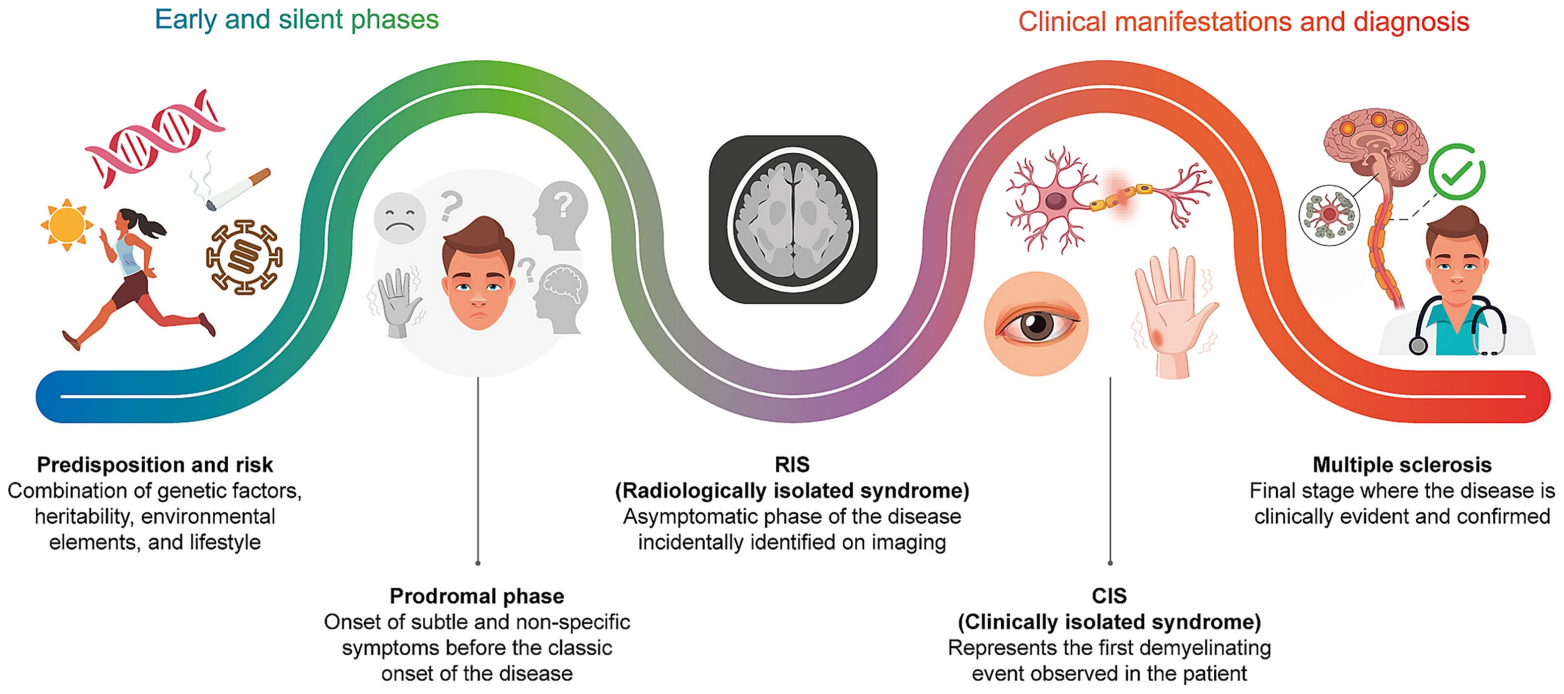
Proposed timeline of MS pathogenesis as a continuum, from the genetic and environmental susceptibility phase through the prodrome, RIS, and CIS to clinically definite disease. CIS, clinically isolated syndrome; MS, multiple sclerosis; RIS, radiologically isolated syndrome.

Within this perspective, the concept of the MS continuum can be understood across three complementary dimensions (Figure 1). First, the biological dimension refers to the evolution of the disease, whereby immune-mediated inflammation and neurodegeneration may begin years before the first clinical manifestations, driven by a complex interplay of genetic susceptibility and environmental exposures. Second, the clinical dimension reflects the temporal trajectory of observable disease manifestations, from early disease manifestations through a first clinical event (CIS) to clinically definite MS. Third, the diagnostic dimension encompasses the advances in imaging and biomarkers that enable the detection and characterization of disease-related processes at progressively earlier stages. Recognizing these complementary but distinct dimensions provides the conceptual framework that guides the structure of this review.

This narrative review synthesizes current evidence across the biological, clinical and diagnostic stages of MS. A literature search was performed using PubMed and Web of Science databases, covering publications up to December 2025. The search strategy combined MeSH terms and free-text keywords related to ‘multiple sclerosis’ and specific topics of interest (e.g., ‘biomarkers’, ‘genetics’, ‘prodrome’), using the Boolean operators AND and OR. Emphasis was placed on systematic reviews, meta-analyses, umbrella reviews, Mendelian randomization studies, which are generally less susceptible to confounding and reverse causation, and large population-based studies when they provided relevant mechanistic or epidemiological support. Large administrative healthcare databases and retrospective analyses were reviewed for the MS prodrome, as these represent the predominant study designs available in the published literature.

Source selection was guided by conceptual relevance, expert judgement and methodological soundness rather than by pre-specified inclusion or exclusion criteria. When multiple sources addressed similar topics, preference was given to the most recent and methodologically robust evidence. Because of the narrative nature of this review, no formal systematic procedure was applied; however, the authors sought to incorporate evidence from different study designs and to reflect areas of both consensus and ongoing uncertainty.

## 2 Genetic and environmental susceptibility phase

Understanding MS as a biological continuum requires considering the earliest stages of disease susceptibility. Long before clinical manifestations appear, a complex interplay between genetic predisposition and environmental factors lays the groundwork for subsequent prodromal and preclinical disease stages.

### 2.1 Genetic susceptibility and heritability

The exact etiology of MS remains unclear; however, one of the most accepted approaches to disease susceptibility is a complex interaction of genetic and environmental factors. The genetic contribution to disease susceptibility is supported by multiple genome-wide association studies (GWAS) that over the past 10 years have identified more than 200 genetic risk variants for MS, 32 of which are located within the human leukocyte antigen (HLA) region (2). The strongest association has been consistently observed in the HLA region, with HLA-DRB1*15:01 being the allele most robustly associated with MS across populations tested. Carrying at least a copy of HLA-DRB1*15:01 roughly triples the risk of developing MS (3). Conversely, HLA-A*02 appears to confer a protective effect (3, 4). Only a small number of non-HLA variants for MS (TNFRSF1A, IL22RA2, TYK2) have been functionally characterized (5–8).

A large-scale analysis by the International MS Genetics Consortium (IMSGC) of genetic data from 47,429 people with MS and 68,374 controls showed that MS susceptibility genes are distributed not only among components of the adaptive immune system, such as T and B cells, but also within innate immune pathways, including natural killer and dendritic cells (2). Interestingly, a significant enrichment of MS-associated genes was identified in purified human microglia, suggesting a potential role for resident CNS immune cells in disease susceptibility. A Mendelian randomization (MR) model further identified a positive association between CD79A expression and MS risk (9), a gene involved in pre-B cell receptor signaling and B-cell activation (10).

The heritability of MS has been estimated at approximately 48%, based on GWAS data (2). This estimate was confirmed by a meta-analysis by Fagnani et al. (11) which showed that roughly half of the overall disease risk is attributable to genetic factors, with the remainder explained by environmental influences (21% shared and non-shared). This study also updated recurrence risk estimates, reporting a 3% risk for full siblings and dizygotic twins and a 24% risk for monozygotic twins, corresponding to concordance rates of 46% and 71%, respectively (11).

Finally, beyond individual risk variants, familial aggregation remains a relevant, though often underexplored, contributor to MS susceptibility. The risk of MS is higher among first-degree relatives of affected individuals (12); however, familial aggregation does not appear to determine the specific clinical phenotype of the disease. The global prevalence of familial MS is estimated at 12%–13% (13, 14), with variability depending on geographic region and latitude (14).

### 2.2 Lifestyle and environmental risk factors

During the initial susceptibility stage, lifestyle and environmental factors interact with genetic predisposition. Among these, Epstein–Barr Virus (EBV) infection, tobacco exposure, obesity (particularly during adolescence), and low vitamin D levels are the most consistently established risk factors. Importantly, EBV infection, smoking, and adolescent obesity interact with HLA risk variants, further increasing MS risk in genetically predisposed individuals (15).

EBV infection represents one of the most robust environmental risk factors for MS. Meta-analyses of observational studies have demonstrated that seropositivity for anti-EBV nuclear antigen (anti-EBNA) antibodies is among the strongest and most consistent associations with MS risk (16). Longitudinal data from the US military cohort showed a markedly increased risk of MS following EBV seroconversion, with serum neurofilament light chain (sNfL) levels rising only after infection, suggesting that EBV precedes the earliest detectable neuroaxonal injury (17). These findings are further supported by case–control studies using preclinical samples, which reported EBV seroreactivity up to a decade before the first objective signs of neuroaxonal damage (18). In addition, high-affinity molecular mimicry between EBNA1 and the CNS protein glial cell adhesion molecule (GlialCAM) provides a plausible mechanistic link between EBV infection and MS pathogenesis (19).

According to an umbrella review of systematic reviews and meta-analyses, smoking is one of the few environmental exposures, together with EBV infection, that shows the most consistent evidence for increased MS risk (20). The effect of smoking is strongly modified by HLA genotype, particularly in individuals carrying HLA-DRB1*15 and lacking HLA-A*02, and this interaction extends to passive smoking exposure (21, 22). The population-attributable fraction of MS due to smoking has been estimated at approximately 18% (23). Evidence regarding the impact of smoking on disability progression is less consistent, although large cohort studies suggest a potential accelerating effect (24).

Regarding vitamin D, umbrella reviews have reported heterogeneous findings from observational studies (20); however, MR analyses have consistently supported a causal relationship between low serum 25-hydroxyvitamin D levels and increased MS risk, independent of other established factors (25–27). A systematic review of MR studies further confirmed low vitamin D levels as a robust risk factor for MS (28). At the molecular level, MS-associated genetic loci are enriched in vitamin D response elements, including within the HLA-DRB1 promoter, reinforcing the biological plausibility of gene–environment interactions involving vitamin D signaling (29). Low sun exposure contributes both directly and indirectly to MS risk and has been associated with both relapse-onset and progressive-onset MS (30, 31).

Obesity, particularly during childhood and adolescence, has emerged as another important and potentially modifiable risk factor. Systematic reviews and MR studies consistently support a likely causal relationship between higher body mass index (BMI) and increased MS risk (32–35). Adolescent obesity shows the strongest effect and interacts with HLA-DRB1*15, further amplifying disease susceptibility (36, 37). From a mechanistic perspective, obesity is associated with chronic low-grade inflammation, characterized by elevated pro-inflammatory cytokines, impaired regulatory T-cell function, and increased accumulation of mature B lymphocytes in visceral adipose tissue, providing a biologically plausible link to MS pathogenesis (38–40). Evidence supporting a role for specific dietary patterns in MS susceptibility remains limited. Observational studies suggest that higher intake of polyunsaturated fatty acids and certain micronutrients may be associated with a reduced risk of a first demyelinating event or MS, but causal inferences remain uncertain (41–43). Accordingly, diet is best regarded as a potential modifier of inflammatory pathways rather than a confirmed preventive intervention.

Epigenetic mechanisms represent an additional interface between environmental exposures and genetic susceptibility. Studies in monozygotic twins discordant for MS have demonstrated differences in DNA methylation and histone modification, supporting a role for non-genetic factors in disease development (44, 45). Among epigenetic regulators, altered DNA methylation at the HLA-DRB1 locus and dysregulation of immune-related microRNAs, particularly miR-155, have been repeatedly implicated, although their causal role remains to be fully established (46–54).

Taken together, these data suggest that integrating susceptibility with environmental and lifestyle exposures into composite risk models may enable identification of individuals at increased MS risk before clinical onset. In this context, the Genes and Environment in Multiple Sclerosis (GEMS) study demonstrated that asymptomatic first-degree relatives with the highest combined risk scores exhibited subtle neurological abnormalities, supporting the concept that MS-related processes may begin well before the clinical threshold is reached (55). This framework provides a rationale for investigating the earliest manifestations of disease and sets the stage for the emerging concept of an MS prodromal phase.

## 3 The prodromal phase in MS

Although well-established in other neurological disorders such as Parkinson’s disease, MS has historically not been considered to have a clearly defined prodromal phase, with disease onset traditionally defined by the first clinical demyelinating event. However, recent evidence from large population-based studies challenges this view, suggesting that MS may be preceded by a phase of subtle and non-specific manifestations spanning approximately 5–10 years before symptom onset and up to 20 years in primary progressive MS, with similar features also described in individuals with RIS (56–69). Data on pediatric populations are emerging but remain limited (56). This phase would thus represent an early detectable window within the disease continuum, preceding both overt clinical onset and formal diagnostic definition.

The prodromal phase is characterized by increased healthcare (HC) utilization, higher prescription rates, and a constellation of recurrent but nonspecific symptoms. Multiple studies from Canada, the UK, Portugal, and Sweden have consistently reported a progressive rise in HC use, psychiatric consultations, and medication prescriptions several years before MS onset (57, 59, 61, 66, 69, 70) (Table 1). Distinct HC utilization trajectories have been identified in the years preceding the first demyelinating event, including frequent consultations with neurologists, neurosurgeons, and ophthalmologists (71). Importantly, higher levels of pre-diagnostic HC utilization have been associated with higher subsequent disability, suggesting that prodromal activity may carry prognostic implications (72). It should be noted, however, that a substantial proportion of these studies rely on large administrative healthcare databases and retrospective analyses, in which symptom patterns and healthcare interactions are reconstructed through diagnostic codes. Such approaches may be susceptible to surveillance bias, diagnostic delay, and misclassification of early neurological manifestations not yet recognized as MS at the time of presentation. Consequently, increased HC utilization prior to diagnosis may, in some cases, reflect unrecognized early disease activity rather than a truly distinct prodromal phase.

**Table 1:** Recent studies on healthcare use in patients with MS before the first demyelinating event or symptom onset.

Author (year)	Country/Design	Review period	MS/Controls	Observation period before disease onset	Healthcare resources use in MS cases compared to controls
Wijnands et al. (2017) (66)	Canada. Matched cohort study, using administrative and clinical databases	April 1984–April 2014	14,428/72,059	5 years before the first demyelinating disease-related claim or clinically reported symptom onset	Hospital admissions: from RR 1.26 (95% CI 1.16–1.36) to 1.78 (1.50–2.10). Physician claims: from 1.24 (1.16–1.32) to 1.88 (1.72–2.07). Prescriptions: from 1.23 (1.06–1.41) to 1.49 (1.41–1.59). Similar patterns for physician claims and prescriptions were observed
Disanto et al. (2018) (59)	UK. Population-based observational nested case–control study.	January 1987–February 2016	10,204 MS/39,448 controls	Up to 10 years before the first record of MS	Median number of GP visits in MS *vs* controls: At 0–2 years before first MS record: 24 (IQR = 13–40) *vs* 11 (IQR = 4–22); *p* < 0.001 At 2–5 years before MS record: 24 (IQR 12–43) *vs* 16 (IQR 7–31); *p* < 0.001 At 5–10 years before the first MS record: 30 (IQR 15–55) vs. 24 (IQR 11–45); *p* < 0.001
Zhao et al. (2020) (69)	Canada. Matched cohort study with data from health administrative hospital, physician, and prescription databases.	1996–2013	4,862 MS/22,649 controls	During the 5 years before the first demyelinating-related claim for MS cases and matched population controls.	MS cases had higher odds than controls of filling a prescription for: Antivertigo preparations (adjusted OR [aOR] 2.48; 95% CI 1.92–3.19). Anti-epileptics (aOR 2.34; 1.90–2.90). Glucocorticoids (aOR 1.76; 1.52–2.03). Urinary anti-spasmodics (aOR 1.72; 1.20–2.46). Muscle relaxants (aOR 1.33; 1.13–1.56).
Jorge et al. (2021) (61)	Portugal. Retrospective review of EHR and compared with national data.		168 MS (no controls)	5 years before MS diagnosis	Mean number of HC use per patient/year: 3.14 ± 2.69, most of them in primary HC (47%). Median number of diagnostic tests per patient: 6 (IQR 7). Median number of drug prescriptions per patient: 6 (IQR 9).
Chertcoff et al. (2023) (57)	Canada. Matched retrospective cohort study using linked administrative and clinical data	January 1, 1996 - December 31, 2013 (administrative cohort) January 1, 1996 - December 31, 2008 (clinical cohort)	6,863/31,865 (administrative cohort) 966/4,534 cases (clinical cohort)	5 years before the first demyelinating claim of cases with MS (administrative cohort) or symptom onset (clinical cohort)	Psychiatric morbidity prevalence ratio was higher in MS patients than controls by 58% in the clinical cohort and 91% in the administrative cohort. In the administrative cohort, HC use was higher for cases in each year pre-MS onset (all 95% CIs > 1): Physician visits: 78% higher in year 5 pre-MS onset and 124% 1 year before. Visits to psychiatrists: 132% higher in year 5 and 146% in year 1. Hospitalizations: 129% higher in year 5 and 197% in year 1. Prescription dispensations: 72% higher in year 5 and 100% in year 1.
Tremlett, et al. (2025) (70)	Canada & Sweden. Population-based health administrative data from Canada and the Swedish MS registry	1991–2020	35,018/136,007 (Ontario) 10,269/51,297 (Sweden)	During the 20 years pre-index date (earliest MS/demyelinating disease-related code)	In Ontario, MS patients showed ≥20% higher annual physician and hospital visit rates for 20 (of 29) years studied, with hospital visits rising to ≥30% in the 7 years pre-index. In Sweden, annual physician visit rates were higher for up to 15 (of 19) years, exceeding 10% in each of the 6 years pre-index date.

aOR, adjusted odds ratio; CI, confidence interval; EHR, electronic health record; GP, general practitioner; HC, healthcare, IQR, interquartile range; MS, multiple sclerosis; RR, rate ratio.

A systematic review (73) and large population-based studies (57, 59, 74–76) have identified psychiatric symptoms such as anxiety, depression, cognitive complaints, and migraine, as common features of the MS prodrome (Table 2). Fatigue has emerged as one of the most prevalent and disabling prodromal symptoms, frequently reported both in individuals with RIS and in the years preceding MS diagnosis (59, 62, 68). Pain syndromes, particularly headache, neck or back pain, limb discomfort and ocular pain, are also frequently reported during the prodromal period and may occur up to a decade before the first demyelinating event (59, 68, 76).

**Table 2:** Recent population-based studies examining symptoms occurring during the 5 years before the first demyelinating event, symptom onset, or MS diagnosis.

Authors (year)	Country/Design	Review period	MS cases/controls	Main symptoms occurring in MS cases compared to controls
Yusuf et al. (2021) (68)	Canada Population-based matched cohort study used linked administrative and clinical databases	1996–2013	6,863/31,865 (before the first demyelinating event/administrative cohort). 966/4,534 (before MS symptom onset/clinical cohort)	Before the first demyelinating event: Fatigue (aOR: 3.37; 95% CI: 2.76–4.10). Sleep disorders (aOR: 2.61; 95% CI: 2.34–2.91). Anemia (aOR: 1.53; 95% CI: 1.32–1.78) Pain (aOR: 2.15; 95% CI: 2.03–2.27) Before MS symptom onset: Sleep disorders (aOR: 1.72; 95% CI: 1.12–2.56) Pain (aOR: 1.53; 95% CI: 1.32–1.76)
Wijnands et al. (2019) (76)	Canada Population-based study linking administrative and clinical data	1984–2014	13,951 MS/66,940 controls (administrative cohort) 3,202 MS/16,006 controls (clinical cohort)	Nervous-system-related hospital and physician encounters were 2–4 times higher in MS cases than controls: Higher rates of healthcare use for the following groups of conditions: Sensory organs (40–128%) Musculoskeletal system (19–70%) Genito-urinary system (17–59%) Skin-related diseases (20–39%) Non-specific ‘ill-defined symptoms and signs’ (16–134%)
Marrie et al. (2019) (75)	Canada Population-based administrative health data of people with IMID and cohorts from general population	1989–2012	3,766 IBD/2,190 MS/6,350 RA. 65,424 controls.	IMID cohort Depression: IRR 1.54; 95% (CI = 1.30–1.84) Anxiety disorders: IRR 1.30; 95% (CI = 1.12–1.51). Similar results for each of the IBD, MS and RA cohorts. Bipolar disorder incidence increased from 3 years before IMID diagnosis (IRR 1.63; 95% CI 1.10–2.40).
Hogg et al. (2018) (80).	Canada Health administrative records	1991–2013	8,669 MS/40,867 controls	Physician encounter for neurologic or sensory disorders was associated with 2 to 5-fold higher odds of MS.
Disanto et al. (2018) (59)	UK Nested case–control study	Not specified	10,204 MS/39,448 controls	Higher pre-index (≤10 years) risk of gastrointestinal, urinary and anorectal disturbances, anxiety, depression, insomnia, fatigue, headache, and pain. MS risk increased with each additional symptom: 0–2 years: OR = 1.51, 95% CI: 1.47–1.55; *p* < 0.001 2–5 years: OR = 1.29, 95% CI: 1.25–1.33; *p* < 0.001 5–10 years: OR = 1.20, 95% CI: 1.15–1.26; *p* < 0.001
Hoang et al. (2016) (74)	DenmarkNested case-control study based on Danish registers.	1999–2009	5,084 MS/24,771 controls	Risk of depression and anxiety in MS patients *vs* controls: OR = 1.4, 95% CI: 1.05–1.88
Cortese et al. (2016) (58)	Norway Nested case–control study based on the historical cohort of men born 1950–1995 who underwent conscription exam.	1950–1995	924 MS/19,530 controls	MS cases scored lower in cognitive performance than controls 2 years before MS symptom onset (*Δ* 0.80, *p* = 0.009). Patients scoring >1 SD below controls had an increased MS risk over the following 2 years (RR: 2.81, 95% CI: 1.52–5.20).

aOR, adjusted odds ratio; CI, confidence interval; IBD, inflammatory bowel disease; IMID, immune-mediated inflammatory diseases; IRR, incidence rate ratio; MS, multiple sclerosis, OR, odds ratio; RA, rheumatoid arthritis; RR, relative risk; SD, standard deviation.

Additional associations include increased susceptibility to infections in the years preceding symptom onset or diagnosis, as well as around the time of diagnosis, potentially reflecting early immune dysregulation (76–78).

Evidence regarding cognitive impairment is less consistent: while some studies report no increase in cognitive complaints prior to diagnosis, others have identified subtle deficits in neuropsychological performance or educational attainment years before MS onset, particularly in primary progressive MS (58, 59, 62, 79). Early autonomic involvement has also been suggested by increased pre-diagnosis healthcare encounters related to urinary, gastrointestinal, and sphincter disorders (59, 68, 80), although these findings remain non-specific. Other reported prodromal features, such as anemia, sleep disorders, and immune-mediated skin diseases, are supported by more limited evidence (68, 77, 81).

Adding biological plausibility to the prodromal hypothesis, biomarker studies suggest that neuroaxonal damage and immune activation may occur before the first clinical event. In a nested case–control study, Bjornevik et al. reported elevated sNfL levels a median of six years prior to clinical MS onset compared with controls (82). Similarly, elevated anti-EBNA1 IgG titers have been detected several years before diagnosis, supporting a role for EBV-mediated immune activation during the preclinical phase (83). The predictive value of these biomarkers as risk factors in isolation remains limited; however, their utility is likely to depend on integration with clinical, imaging, and molecular data within composite risk models.

Collectively, these findings support the possibility of an identifiable prodromal phase in MS with partially distinct profiles across relapsing and progressive phenotypes (67). Nevertheless, it is important to acknowledge that many of the currently identified prodromal features represent statistical associations observed at the population level, and their predictive value for identifying individuals who will later develop MS remains limited. The non-specific nature of prodromal symptoms, combined with the methodological constraints inherent to administrative database studies, limits their usefulness as isolated screening tools.

## 4 Radiologically isolated syndrome: a pre-clinical stage of MS or part of a broader prodromal phase?

RIS refers to the incidental detection of brain or spinal cord MRI abnormalities suggestive of MS in individuals without clinical symptoms attributable to inflammatory demyelination. RIS represents a stage primarily defined at the diagnostic level, while reflecting underlying biological activity in the absence of clinical manifestations. Increasing interest in the earliest stages of MS has raised the question of whether RIS represents a distinct preclinical entity or, in some individuals, part of a broader spectrum of disease manifestations preceding clinical onset.

The 2024 revision of the McDonald diagnostic criteria (84) introduces an important conceptual advance by recognizing that MS may now be diagnosed in asymptomatic individuals with RIS who meet dissemination in space (DIS) and dissemination in time (DIT) criteria on MRI, once alternative explanations have been carefully evaluated and excluded. Nevertheless, the clinical implications of diagnosing MS in asymptomatic individuals remain a matter of debate within the neurological community. While the revised criteria expand the incorporation of radiological and biomarker evidence into the diagnostic framework, they do not imply that RIS should routinely be reclassified as MS; rather, they reflect an evolving understanding of MS as a biological, clinical and diagnostic continuum, and acknowledge the prognostic significance of subclinical neuroinflammation detectable through paraclinical markers.

In the original description of RIS, headache was the most frequent indication for MRI acquisition (85), a finding confirmed in subsequent analyses (86, 87). Less frequently, mood disorders and pain were also documented as MRI triggers. Although these symptoms are non-specific, they overlap with those described during the MS prodromal phase. In some cases, particularly when demyelinating lesions involve eloquent CNS regions, they may reflect early functional manifestations of subclinical disease activity (87). Nevertheless, such symptoms have not been shown to predict conversion from RIS to clinically definite MS (63).

Most population-based studies demonstrating increased HC utilization prior to MS diagnosis lack MRI data, which limits their ability to specifically evaluate RIS. To address this gap, Lebrun-Frenay et al. analyzed HC use in the three years preceding RIS diagnosis and found that approximately half of the individuals exhibited increased healthcare contacts, predominantly related to headaches, whereas the remainder had no recorded interactions prior to their index MRI (88). Although these findings did not support the existence of a distinct prodromal phase uniformly preceding RIS, the overlap with features described prior to clinically manifest MS suggests heterogeneity within the RIS population (89).

RIS is increasingly regarded as part of the diagnostic and biological MS continuum, with approximately 50% of individuals developing a first clinical event, thereby crossing the threshold of the clinical continuum, within 10 years of follow-up (63). This evidence suggests that some individuals may already be within the prodromal stage at the time of RIS diagnosis. Established risk factors for clinical conversion include younger age, CSF oligoclonal bands, elevated k-free light chain (k-FLC) index, spinal cord or infratentorial lesions, and ongoing radiological activity during follow-up (63, 65, 90). CSF NfL levels have also shown prognostic value for clinical conversion (91). In contrast, serum biomarkers, including sNfL, have not yet shown sufficient predictive accuracy to guide individual risk stratification in RIS (17).

Defining the relationship between RIS and the MS prodrome remains challenging due to the non-specific nature of prodromal symptoms and the high prevalence of overlapping comorbidities, such as depression, autonomic dysfunction, and recurrent infections, which are common in other immune-mediated conditions (63, 92). This creates a dual challenge: distinguishing the MS prodrome from that of other autoimmune disorders (88, 92), while avoiding misattribution of nonspecific symptoms to early MS. In this context, reports indicating that a substantial proportion of patients presenting with a first demyelinating event had experienced unrecognized suggestive symptoms in the preceding years are particularly relevant (93).

In summary, RIS occupies a pivotal position within the biological and diagnostic MS continuum, but its diagnostic interpretation remains context-dependent. While some individuals with RIS may represent early stages of disease, the heterogeneity of clinical and biological profiles highlights the need for imaging and fluid biomarkers to better define individual risk. At present, RIS represents the earliest stage at which disease-modifying interventions have been formally evaluated. In the ARISE (94) and TERIS trials (95), dimethyl fumarate and teriflunomide reduced the risk of a first clinical event by 82% and 72%, respectively, highlighting the plausibility of early intervention in RIS, while underscoring the need for more evidence to guide treatment decisions in this population.

## 5 Clinically isolated syndrome

CIS refers to a first episode of neurological symptoms caused by CNS inflammation and demyelination, lasting at least 24 h, in the absence of alternative diagnoses and in individuals not previously known to have MS (96). It typically affects young adults and most commonly involves the optic nerves, brainstem, or spinal cord (97). At this stage, disease expression becomes clinically overt, marking a key transition in the clinical trajectory and closely linked to the diagnostic framework.

Epidemiological studies indicate that optic neuritis accounts for approximately 30–50% of first demyelinating events, followed by spinal cord syndromes (30–45%), brainstem or cerebellar syndromes (10–25%), and hemispheric or multifocal cerebral presentations (20–25%), depending on the cohort studied (98, 99). Advances in MRI and CSF analysis have progressively blurred the traditional diagnostic boundary between CIS and clinically definite MS, such that many individuals presenting with a first clinical event now fulfill diagnostic criteria for MS shortly after onset (96). This evolution in the diagnostic framework reflects a deeper understanding of the underlying disease processes: by the time a first clinical event occurs, subclinical disease activity, including silent lesion accumulation and neuroaxonal injury, may have been present for months or years. CIS may therefore be better understood not only as a transient precursor, but as a clinically overt stage within a broader biological, clinical and diagnostic continuum.

Key pathophysiological mechanisms active in CIS include early immune dysregulation, focal demyelination, and neuroaxonal injury. Evidence for an early neurodegenerative component comes from a meta-analysis comparing CSF biomarkers across MS phenotypes and healthy controls, which demonstrated elevated levels of NfL, chitinase-3-like protein 1 (CHI3L1), and total tau (t-tau) in patients with CIS (100) (Table 3). These findings indicate that axonal damage and glial activation are already present at the first clinical manifestation. In parallel, a systematic review reported increased levels of inflammatory CSF biomarkers, including intrathecal IgG synthesis and multiple pro-inflammatory cytokines, in individuals with CIS, which were associated with faster conversion to MS, higher relapse rates, and greater long-term disability. (101). Together, these data support the coexistence of inflammatory and neurodegenerative processes from the earliest clinical stages.

**Table 3:** Summary of systematic reviews and/or meta-analyses on CIS from the last 5 years in relation to the MS continuum concept.

Authors (year)	Population	Features assessed	Key findings	Relevance to MS continuum approach
Al-Namaeh et al. (2021) (109) (Systematic review and meta-analysis)	CIS	Early DMTs (IFN, glatiramer) and optic-neuritis-specific treatments	Early treatment reduced the risk of conversion to MS	Early intervention may modify disease trajectory and supports the concept of an early and potentially modifiable disease continuum beginning at CIS.
López-Gómez et al. (2023) (102) (Systematic review)	CIS	MRI (DIS/DIT), and CSF biomarkers (OCB, k-FLC, NfL, CHI3L1)	k-FLC; NfL, lipid-specific IgM and CHI3L1 strongly predict early conversion.	Detectable immune and neurodegenerative markers at CIS are associated with progression.
Momtazmanesh et al. (2021) (100) (Systematic review and meta-analysis)	CIS, RRMS, PMS vs. controls	CSF biomarkers (NfL, CHI3L1, t-tau)	CIS cases had higher levels of CSF biomarkers compared with control.	Supports the presence of neurodegeneration and glial activation from the CIS stage onward.
Temmerman et al. (2023) (101) (Systematic review)	CIS, RIS, RRMS, SPMS	Inflammatory CSF markers	Higher levels of inflammatory markers in CIS predict faster conversion, relapses and/or disability; may persist despite DMTs.	Supports the presence of early and persistent innate immune system-driven activity from CIS through MS.

CHI3L1, Chitinase-3-like protein 1; CIS, clinically isolated syndrome; CSF, cerebrospinal fluid; DIS, dissemination in space; DIT, dissemination in time; DMTs, disease-modifying therapies; IFN, interferon; IgM, immunoglobulin M; k-FLC, kappa free light chain; MRI, magnetic resonance imaging; MS, multiple sclerosis; NfL, neurofilament light chain; OCB, oligoclonal bands; PMS, progressive multiple sclerosis; RIS, radiologically isolated syndrome; RRMS, relapsing–remitting multiple sclerosis; SPMS, secondary progressive multiple sclerosis; t-tau, total Tau protein.

Biomarkers measurable at the CIS stage may contribute to risk stratification in individuals presenting with a first demyelinating event, although their predictive value at the individual level remains variable. For example, CSF k-FLC index and CSF NfL levels have been associated with an increased likelihood of conversion to MS within short-term follow-up in cohort studies (102). The presence of oligoclonal bands (OCBs) in CSF reflects persistent intrathecal IgG synthesis and ongoing immune activation even before a clinical diagnosis of MS is established (102). These findings highlight the coexistence of inflammatory and neuroaxonal processes at the CIS stage.

According to the 2017 McDonald diagnostic criteria, a patient presenting with CIS can be diagnosed with MS if MRI findings demonstrate both DIS and DIT, or if CSF-OCBs are present, as their detection may substitute for DIT (103) (Table 4). This revision enabled earlier diagnosis in patients previously classified as CIS, particularly when asymptomatic MRI lesions or intrathecal IgG synthesis were detected (104, 105). Notably, the 2024 McDonald revision further refines this approach by incorporating RIS under specific conditions, potentially reclassifying some individuals at first clinical presentation as already having MS (84). Historical cohort studies reported that 50%–75% of untreated individuals with CIS and MRI lesions converted to MS within two years, and more than 85% progressed to relapsing or progressive disease over longer follow-up (106–108).

**Table 4:** Evolution of the McDonald diagnostic criteria for multiple sclerosis (2001–2024).

Diagnostic domain	2001	2005	2010	2017	2024
Dissemination in space (DIS)	Primarily clinical; MRI supportive using Barkhof–Tintoré criteria (≥3/4)	Barkhof–Tintoré MRI criteria (≥3/4); spinal cord lesion may replace infratentorial or enhancing brain lesion	≥1 T2 lesion in ≥2/4 regions (periventricular, juxtacortical, infratentorial, spinal cord); symptomatic lesions excluded in brainstem/spinal cord syndromes	≥1 T2 lesion in ≥2/4 regions; symptomatic and asymptomatic lesions both count	≥1 lesion in ≥2/5 regions: optic nerve, cortical/juxtacortical, periventricular, infratentorial, spinal cord. PPMS: ≥2 spinal cord lesions are sufficient for DIS
Dissemination in time (DIT)	Second clinical attack ≥30 days apart, or MRI (≥3 months)	Gd + lesion ≥3 months after onset (not symptomatic site) or new T2 lesion vs. reference MRI ≥ 30 days	New T2 and/or Gd + lesion at any time or simultaneous asymptomatic Gd+/Gd − lesions	Same as 2010; CSF OCBs may substitute for DIT if DIS is met	DIT not mandatory in specific diagnostic scenarios (retained to increase specificity)
CSF biomarkers	Supportive only (OCBs and/or IgG index)	Supportive only	Supportive only	CSF-restricted IgG OCBs substitute for DIT	OCBs or k-FLC index define CSF positivity (interchangeable)
Primary progressive MS (PPMS)	≥1 year progression + DIS and DIT evidence; CSF strongly recommended	≥1 year progression + 2 of 3: positive brain MRI, ≥2 spinal cord lesions, positive CSF	Same requirements as 2005 (≥1 year progression + 2 of 3 criteria)	Same as 2010	≥12 months progression; ≥2 spinal cord lesions explicitly accepted as evidence of DIS in PPMS
Radiologically isolated syndrome (RIS)	Not defined	Not considered MS	Not considered MS	Not considered MS	RIS may be diagnosed as MS if DIS + DIT or CSF positivity; CVS select-6 is sufficient to support DIS
Supportive MRI markers	Not included	Not included	Not included	Not included (research only)	CVS and PRL accepted as supportive markers in selected scenarios
CVS definition/threshold	—	—	—	—	Select-6 rule: ≥6 white matter lesions with CVS; if <10 WM lesions, the majority must be CVS+
PRL definition/threshold	—	—	—	—	PRL-positive = ≥1 paramagnetic rim lesion
High-specificity diagnostic shortcuts	—	—	—	—	≥4/5 typical regions, MS diagnosed without additional requirements (if no better explanation). If lesions are confined to one region, CVS select-6 or ≥1 PRL plus DIT or CSF positivity is sufficient

CSF, cerebrospinal fluid; CVS, central vein sign; DIS, dissemination in space; DIT, dissemination in time; Gd+/Gd−, gadolinium-enhancing/non–gadolinium-enhancing lesions; IgG, immunoglobulin G; k-FLC, kappa free light chain; MRI, magnetic resonance imaging; MS, multiple sclerosis; OCBs, oligoclonal bands; PPMS, primary progressive multiple sclerosis; PRL, paramagnetic rim lesion; RIS, radiologically isolated syndrome; WM, white matter.

Adapted from McDonald WI et al. (170), Polman CH et al. (171), Thompson AJ et al. (103), Montalban X et al. (84), Cohen JA. (169), and Agrawal M et al. (168).

The management of CIS has evolved in parallel with these diagnostic advances. Randomized clinical trials demonstrated that early initiation of disease-modifying therapies (DMTs), such as interferon-beta and glatiramer acetate, delays conversion to MS and reduces relapse rates, with favorable long-term safety profiles (109). More recently, real-world evidence and emerging trial data have supported consideration of earlier use of high-efficacy therapies in selected patients with unfavorable prognostic features (110, 111).

Risk stratification at the CIS stage increasingly incorporates clinical, radiological, and biomarker information, including lesion burden and location, CSF OCBs, k-FLC index, and NfL levels in CSF and serum (112). While concerns regarding overtreatment in low-risk individuals remain (113), expert consensus highlights the potential consequences of undertreatment, particularly in light of accumulating evidence of silent disease activity and the availability of safer therapeutic options and improved monitoring strategies (113, 114). To optimize early treatment decisions, prognostic tools are under development to integrate clinical, imaging, and biomarker data into composite risk scores that may support risk stratification and clinical decision-making (115, 116).

In summary, the CIS stage represents a key opportunity for early therapeutic intervention, although treatment decisions should be guided by individualized risk assessment and the current evidence base.

## 6 Biomarkers: what is their role across the MS continuum?

Over the past three decades, the search for biomarkers in MS has been extensive. However, only a limited number have been validated and are currently used in clinical practice. In this section, we focus on selected biomarkers that have shown potential utility across the biological, clinical, and diagnostic continuum.

Some of these biomarkers, such as sNfL, glial fibrillary acidic protein (GFAP), and immunological and viral markers, reflect underlying disease processes, including neuroaxonal injury, reactive astrogliosis, and immune dysregulation. CSF biomarkers such as OCBs, the k-FLC index, and lipid-specific IgM bands, support early disease recognition and risk stratification (117–120). Together with MRI-based parameters and optical coherence tomography (OCT) as a surrogate for axonal degeneration, these biomarkers collectively span the biological and diagnostic dimensions of the continuum, capturing different aspects of disease activity from preclinical stages through to clinically definite MS, although their specificity and clinical applicability remain variable.

### 6.1 Magnetic resonance imaging

MRI remains the gold standard for the evaluation of suspected MS. In individuals with RIS and CIS, MRI can reveal demyelinating lesions before or at the time of the first clinical manifestation, providing objective evidence of subclinical disease activity (121).

Beyond conventional lesion detection, advanced MRI markers reflecting characteristic MS pathology have gained increasing relevance in diagnostic and prognostic assessment. These include the central vein sign (CVS), which indicates the perivenular origin of white matter lesions, and paramagnetic rim lesions (PRLs), which reflect chronic active inflammation at lesion borders (122, 123). In the 2024 McDonald revision, CVS and PRLs are now recognized as typical MS features, although they are not mandatory for diagnosis and must be interpreted within the broader radiological and clinical context (84).

A systematic review and meta-analysis including over 100 studies demonstrated robust associations between T1 and T2 lesion volumes, gray matter and white matter atrophy, and disability (as measured by the Expanded Disability Status Scale, EDSS) (Table 5) (124). These results are consistent with a more recent and substantially larger meta-analysis including more than 38,000 patients, which confirmed that higher lesion burden and more pronounced regional and global atrophy correlate with disability indices and MRI markers of disease severity (125). Together, these data reinforce the utility of MRI-derived markers as indicators of disease activity and long-term clinical outcomes.

**Table 5:** Summary of systematic reviews and/or meta-analyses on biomarkers from the last 5 years in relation to the MS continuum concept.

Authors (year)	Population	Features assessed	Key Findings	Relevance to MS continuum approach
Magnetic resonance imaging (MRI)				
Nabizadeh F. et al. (2024) (124) (Systematic review and meta-analysis)	MS (unspecified phenotypes)	MRI lesion and atrophy metrics	T1/T2 lesion burden and gray/white matter atrophy correlate with disability	MRI measures reflect disease burden and may help monitor MS progression.
Mirmosayyeb O. et al. (2024) (125) (Systematic review and meta-analysis)	MS (mixed phenotypes)	MRI lesion metrics and brain volumes	Cortical and white matter lesion burden and reduced regional/global volumes correlate with EDSS	Supports the use of MRI metrics as quantitative markers across the MS continuum
Zivadinov R. et al. (2025) (126) (Systematic review)	MS (unspecified phenotypes)	Global brain atrophy measures	Faster atrophy progression predicts EDSS worsening.	Atrophy reflects cumulative neurodegeneration across the disease course.
Rocca MA et al. (2023) (127) (Narrative review with systematic approach)	MS (unspecified phenotypes)	Advanced MRI	Microstructural and gray matter changes detectable beyond conventional MRI.	Enhances understanding of MS pathology across disease stages.
Krajnc N et al. (2021) (128) (Systematic review)	MS (unspecified phenotypes)	Global and regional cortical and subcortical atrophy, number of iron rim lesions, and cervical spinal cord average CSA	Atrophy measures are reproducible but technically limited.	Atrophy is a potential marker for progressive disease.
Optical coherence tomography (OCT)				
El Ayoubi et al. (2024) (129) El Ayoubi et al. (2025) (130) (Systematic review and meta-analysis)	MS (unspecified phenotypes)	pRNFL, macular RNFL, GCIPL, INL, ONL	Retinal thinning correlates with age, duration, EDSS, DMT use.	OCT detects neurodegeneration across MS stages.
Mirmosayyeb et al. (2023) (131) (Systematic review and meta-analysis)	RRMS	RNFL and GCIPL thickness	RNFL thinning correlates with cognitive decline.	Retinal atrophy mirrors MS-related cognitive impairment.
Mohammadi et al. (2023) (132) (Systematic review and meta-analysis)	MS (unspecified patient phenotypes)	OCT-A microvascular measures	Retinal vascular changes noted; data heterogeneous.	Suggests vascular contribution to MS pathology.
Cerebrospinal fluid (CSF) oligoclonal bands (OCBs)				
Fonderico et al. (2021) (135) (Meta-analysis)	CIS, RRMS, and PPMS	OCBs and ITMS	OCB-positive and ITMS-positive patients have higher relapse risk	Early CSF markers predict disease activity from onset.
Hegen H et al. (2023) (134) (Systematic review and meta-analysis)	CIS or MS vs. controls	CSF k-FLC vs. OCB	k-FLC has diagnostic performance similar to OCBs.	Reflects ongoing immune activity from CIS to MS.
Liampas A et al. (2025) (172) (Systematic review and meta-analysis)	RRMS on NTZ	OCB dynamics	NTZ reduces OCBs	OCBs may change over time and may reflect treatment response.
Naseri et al. (2021) (173) (Systematic review and meta-analysis)	Late-onset MS	OCB/IgG index	OCBs remain present in older-onset MS.	Immune activation persists in late-onset disease.
Serum neurofilament light chain (sNfL)				
Freedman et al. (2025) (139) (Systematic review)	MS (mixed subtypes)	sNfL	High sNfL predicts relapses and progression.	sNfL reflects ongoing disease activity.
Ning et al. (2022) (140) (Systematic review and meta-analysis)	MS (including CIS) vs. controls	sNfL	sNfL levels are higher in MS and are associated with faster progression.	sNfL may help differentiate disease stages and severity.
Williams et al. (2021) (143) (Systematic review)	PPMS and SPMS	NfL (serum/CSF)	NfL linked to inflammation and atrophy.	Common biology underlies relapsing and progressive forms.
Immune and viral markers				
Bose et al. (2024) (146) (Systematic review)	MS (various subtypes)	Serum EBV antibodies (EBNA1, VCA)	EBV titers vary; transient increase with MRI activity.	EBV not reliable as a progression biomarker
Jacobs et al. (2020) (147) (Meta-analysis)	MS vs. controls (Unspecified subtypes)	EBV serology (EBNA1)	High EBV titers increase MS risk, especially with HLA-DRB1*1501	Supports EBV in MS risk, not progression.
Khalesi Z et al. (2023) (148) (Systematic review and meta-analysis)	MS vs. controls (Unspecified subtypes)	Seroprevalence of herpes viruses (EBV, VZV, HSV-1/2, CMV, HHV-6/7/8)	EBV, VZV, HHV-6 more common in MS.	Chronic infection may contribute to MS onset.
Polygenic risk scores				
Shams et al. (2023) (149) (Systematic review)	European ancestry	MS susceptibility PRS	PRS predicts MS risk and phenotype.	Suggests that genetic susceptibility may influence the MS disease spectrum and progression.
Vasileiou et al. (2022) (150) (Meta-analysis)	MS (Unspecified subtypes)	Vitamin D PRS	No link between vitamin D PRS and outcomes.	Genetic risk may matter more than environmental modifiers.
Composite risk algorithms				
Brown et al. (2020) (151) (Systematic review)	RRMS	Composite risk models	Few tools predict well; often require unavailable data.	Better models needed for early-stage risk prediction.
Reeve et al. (2023) (152) (Systematic review)	CIS, RRMS, SPMS	Prognostic models	Tools vary in accuracy and adoption.	

CIS, clinically isolated syndrome; CMV, cytomegalovirus; CSA, cross-sectional area; DMT, disease-modifying therapy; EBNA1, Epstein–Barr nuclear antigen 1; EBV, Epstein–Barr virus; EDSS, expanded disability status scale; GCIPL, ganglion cell-inner plexiform layer; HHV-6, human herpesvirus 6; HHV-7, human herpesvirus 7; HHV-8, human herpesvirus 8; HSV-1, herpes simplex virus type 1; HSV-2, herpes simplex virus type 2; IgG, immunoglobulin G; INL, inner nuclear layer; ITMS, intrathecal IgM synthesis; k-FLC, kappa free light chain; MRI, magnetic resonance imaging; MS, multiple sclerosis; NfL, neurofilament light chain; NTZ, natalizumab; OCT, optical coherence tomography; OCT-A, optical coherence tomography angiography; OCBs, oligoclonal bands; ONL, outer nuclear layer; pRNFL, peripapillary retinal nerve fiber layer; PPMS, primary progressive multiple sclerosis; PRS, polygenic risk score; RNFL, retinal nerve fiber layer; RRMS, relapsing–remitting multiple sclerosis; sNfL, serum NfL; SPMS, secondary progressive multiple sclerosis; T1, T1-weighted; T2, T2-weighted; VCA, viral capsid antigen; VZV, varicella-zoster virus.

Importantly, not all atrophy metrics perform similarly. The predictive value of global brain volume considered in isolation is less consistent, as highlighted by a 2025 systematic review conducted by Zivadinov et al. (126), which found heterogeneous associations between global atrophy and EDSS once confounding variables, such as lesion burden and age, were accounted for. In contrast, regional measures, particularly deep gray matter structures such as the thalamus, have demonstrated stronger and more reproducible associations with physical disability and cognitive impairment (125, 127). Thalamic atrophy emerges early in the course of disease, including in CIS and even RIS, and has become one of the most sensitive MRI correlates of neurodegeneration.

Beyond conventional metrics, advanced MRI techniques such as quantitative gray matter atrophy measurement, diffusion tensor imaging, susceptibility-based techniques, and longitudinal volumetric approaches for detecting slowly expanding lesions (SELs) provide additional insights into chronic inflammatory activity and tissue loss (127). These techniques support a more nuanced characterization of MS pathology, integrating inflammatory and neurodegenerative components.

The integration of MRI phenotyping into modern classification systems reflects the dynamic nature of MS. Furthermore, the reproducibility of brain atrophy measurements across scanners and platforms, despite unavoidable methodological variability, supports their potential role as surrogate markers of disease progression, although methodological variability and standardization remain important challenges (128).

### 6.2 Optical coherence tomography

OCT has gained interest as a non-invasive method for assessing retinal neuroaxonal integrity in MS and in high-risk individuals. A significant thinning of the peripapillary retinal nerve fiber layer (pRNFL) and ganglion cell-inner plexiform layer (GCIPL) has been reported in MS patients compared with controls (Table 5) (129). These changes were observed across disease stages, including in patients without a history of optic neuritis, and were inversely correlated with disease duration, EDSS, and treatment exposure, suggesting that retinal changes may reflect broader neurodegenerative processes (130). A recent meta-analysis demonstrated that reduced RNFL thickness is significantly associated with impairments in cognitive speed, auditory attention, and memory, supporting the potential of OCT as a surrogate marker of both structural and functional progression (131). Moreover, OCT angiography has revealed microvascular abnormalities in patients with MS, pointing to a possible vascular contribution to neurodegeneration (132). In particular, inner retinal layer thinning measured by OCT in individuals with RIS has been shown to be an independent risk factor for conversion to clinically definite MS, supporting the concept that subclinical neuroaxonal damage in the visual pathway may represent an early stage of MS-related neurodegeneration (133).

### 6.3 Cerebrospinal fluid oligoclonal bands

The detection of CSF-restricted IgG OCBs, defined as two or more IgG bands in CSF not detected in serum, remains a key diagnostic marker of MS. OCBs were incorporated into the 2017 McDonald criteria as evidence that can substitute for dissemination in time. Their presence in CIS and RIS is associated with an increased risk of conversion to clinically definite MS (Table 5) (134).

A meta-analysis of CSF studies showed that OCB-positive CIS patients have more than twice the risk of a second relapse compared to OCB-negative individuals, and that intrathecal IgM synthesis (ITMS) further increases this risk to 3.6-fold (135). In particular, the detection of lipid-specific IgM bands, despite technical challenges, has been associated with a more aggressive disease course and differential responses to high-efficacy therapies (136).

In parallel, the k-FLC index has emerged as a quantitative biomarker of intrathecal immunoglobulin synthesis, reflecting the production of IgG, IgA, and IgM, and has been incorporated into the 2024 McDonald criteria as an alternative to OCBs (134). Beyond its diagnostic utility, several longitudinal studies suggest that higher k-FLC index values are associated with an increased risk of conversion to MS and greater long-term disability, with prognostic performance comparable to that of OCBs across several cohorts (137). However, no universal prognostic cut-off has been established.

Overall, these findings highlight k-FLC as a complementary marker to IgG and IgM OCBs, contributing to refinement of early risk stratification in MS.

### 6.4 Serum neurofilament light chain and glial biomarkers

NfL is a cytoskeletal protein released into CSF and blood following axonal injury. With the development of highly sensitive assays, NfL can now be reliably measured in serum (sNfL), providing a minimally invasive biomarker for neuroaxonal damage in MS (Table 5) (138, 139). Elevated sNfL levels have been observed before and during clinical relapses, have been reported to correlate with the appearance of new MRI lesions, and to be associated with brain atrophy and disability progression in patients with MS (139). Notably, low or age-adjusted normal sNfL values have been linked to a low short-term risk of inflammatory disease activity, including absence of clinical and radiological activity (“no evidence of disease activity”, NEDA), suggesting a potential role in risk stratification across disease stages (139). Meta-analyses have shown that sNfL levels are significantly higher in MS patients compared with healthy controls, and may be modestly higher in progressive phenotypes when inflammatory activity is present (140). In contrast, in non-active progressive disease, sNfL concentrations often remain within low to intermediate ranges, reflecting the biomarker’s predominant sensitivity to inflammation-driven axonal injury rather than to progression independent of relapse activity (141). Consistent with this, elevated sNfL levels are associated with accelerated disability accumulation primarily when progression is accompanied by ongoing inflammatory activity, whereas their ability to predict progression in the absence of inflammation is limited (142). Accordingly, sNfL responses to putative neuroprotective therapies in progressive MS have been variable and generally modest, highlighting its sensitivity to acute or subacute axonal damage rather than chronic neurodegeneration (143).

Another promising biomarker is GFAP, which reflects astrocytic activation and glial pathology. Unlike sNfL, which predominantly captures inflammatory-driven axonal injury, serum GFAP (sGFAP) may better reflect astrocytic activation and the glial processes associated with progression independent of relapse activity (PIRA). A recent multicenter study demonstrated that sGFAP at diagnosis was associated with long-term disability, particularly in patients with low sNfL but elevated sGFAP, highlighting its value in identifying silent progression (144). In a large real-world cohort, elevated sGFAP predicted PIRA, future need for gait aid, and conversion to SPMS, supporting its role as a marker of chronic, non-relapsing disease activity (145). These findings are consistent with prior and subsequent studies linking higher sGFAP levels to disability progression and PIRA in both relapsing and progressive MS.

Taken together, sNfL and sGFAP provide complementary information and may help distinguish between relapse-driven and progression-driven pathology, with potential implications for risk stratification and clinical decision-making.

### 6.5 Immune and viral markers

While immune and viral exposures, particularly EBV, are well-established risk factors for MS development, their utility as biomarkers for disease monitoring and progression remains limited. A systematic review found that EBV-specific antibody titers, especially against EBNA1 and viral capsid antigen (VCA), did not consistently correlate with clinical relapses or MRI activity, although transient increases were occasionally observed during periods of new MRI lesion formation (Table 5) (146). Recent studies have confirmed that anti-EBNA1 IgG, including responses to high-affinity epitopes such as EBNA1 381–420, is consistently associated with MS susceptibility and may rise during the preclinical phase; however, current evidence suggests that it does not reliably reflect disease activity or treatment response once MS is established (147).

Similarly, seropositivity for other herpesviruses, including human herpesvirus 6 (HHV-6), varicella-zoster virus (VZV), herpes simplex virus 1 (HSV-1), and cytomegalovirus (CMV), has been linked to increased MS susceptibility in some cohorts, but none have demonstrated consistent associations with disease course, progression, or treatment response (148). Viral DNA detection in blood or CSF has likewise not shown diagnostic or prognostic utility in routine clinical practice.

Overall, while these immune and viral markers provide important insights into MS initiation and may reflect early immune priming or reactivation events, they currently lack sufficient specificity and predictive value to support their routine use in disease monitoring or prognostic stratification. The precise mechanisms by which these viral agents contribute to MS pathogenesis (whether through molecular mimicry, chronic immune activation, or latent infection of CNS-resident cells) remain incompletely understood.

### 6.6 Polygenic risk scores

Advances in genomics have substantially improved our understanding of genetic susceptibility to MS, particularly through the development of polygenic risk scores (PRS). These scores integrate the cumulative effects of numerous genetic variants, largely within the HLA region and other immune-related loci, to estimate inherited genetic susceptibility to MS. Although several studies have explored whether PRS are also associated with clinical phenotypes, disease course, or progression, current evidence suggests that their predictive value beyond susceptibility, particularly at the individual level, remains limited (Table 5) (149). Consistent with this, a meta-analysis evaluating a vitamin D-related PRS found no clear association with relapse rates, disability progression, or MRI activity (150), suggesting that, while genetic predisposition contributes to overall susceptibility, its role in risk stratification beyond population-level analyses remains uncertain.

### 6.7 Composite risk algorithms

The complexity of MS requires multidimensional tools to support the assessment of disease course. Recent systematic reviews have evaluated a range of composite risk models integrating demographic, clinical, MRI, and biomarker data (Table 5) (151, 152). Although several tools, such as the Bayesian Risk Estimate for MS (BREMS) and Clinically Definite MS (CDMS) scores, have shown potential, their clinical implementation is limited by data availability, methodological heterogeneity, and the need for external validation. Nonetheless, these composite models represent an important step toward improved risk stratification in MS.

## 7 Future directions and expert perspective: towards a predictive, preventive, and personalized MS model

### 7.1 Embracing the continuum: implications for early intervention

Adopting a continuum model of MS, from genetic susceptibility and environmental exposures through early disease manifestations, RIS, and CIS, is reshaping how risk is assessed and disease prevention is envisioned. Central to this paradigm is the need to improve risk stratification in individuals at higher risk before the first clinical demyelinating event, supporting tailored monitoring strategies and informing research into preventive interventions. In this context, a large population-based study demonstrated that a high PRS is associated with an increased risk of MS (153), while a complementary work has shown that PRS not only predicts MS susceptibility but also relates to specific clinical phenotypes and structural markers of tissue injury, such as thalamic atrophy (149). More recently, combining PRS with family history and viral exposures (EBV serostatus) have been shown to improve risk stratification beyond clinical factors alone (154).

These findings suggest the potential feasibility of composite algorithms integrating genetic, environmental, imaging, and biomarker data to improve risk stratification before clinical onset. Prospective validation in large cohorts remains an important next step.

### 7.2 Stratifying risk and redefining intervention thresholds

The 2024 McDonald criteria have expanded the MS diagnostic framework to include selected individuals previously classified as RIS, provided alternative explanations are excluded (84). This change may help reduce ambiguity in early treatment decisions, as individuals who meet the 2024 McDonald criteria will increasingly be considered for management as MS and may be evaluated for DMTs, even if they were previously classified as RIS. Nonetheless, it is worth reiterating that the clinical implications of diagnosing MS at this stage remain a matter of debate and, therefore, treatment decisions in these very early stages require individualized risk–benefit assessment, given the limited evidence base and the lack of full consensus.

For true RIS cases that do not fulfill diagnostic thresholds, current expert opinion generally favors risk-based stratification to guide monitoring intensity. High-risk features such as spinal cord lesions, infratentorial involvement, CSF OCB or k-FLC positivity, and elevated sNfL levels may support closer follow-up and earlier intervention if conversion occurs (155). There is currently insufficient clinical evidence to support the initiation of therapy during early asymptomatic stages before any diagnostic criteria are met. Instead, the goal should be to refine composite risk models that integrate genetic, imaging, serological, and symptomatic features to guide monitoring and, in some cases, inform decisions regarding early intervention. Such models must also address ethical issues: how and when to communicate elevated risk to asymptomatic individuals, and how to balance the benefits of early treatment against possible overtreatment.

### 7.3 Biomarker identification and validation

The identification and validation of novel biomarkers for possible prodromal and preclinical stages of MS remains an important research priority. Advances in next-generation sequencing (NGS), proteomics and microRNA profiling in blood and CSF hold promise for identifying disease-associated molecular signatures that may precede overt neuroinflammation or demyelination (156, 157). These approaches may contribute to a more detailed characterization of the earliest biological processes underlying MS.

In parallel, advanced neuroimaging techniques, such as translocator protein-positron emission tomography (TSPO-PET), which detects microglial activation, and ultra-high-field MRI (≥7 T) allow *in vivo* visualization of cortical lesions, meningeal inflammation, and other subtle structural abnormalities not detectable with conventional imaging (158, 159). These modalities may serve as noninvasive tools to identify patients at risk before clinical symptoms appear and could play a key role in future preventive or neuroprotective trials.

Ultimately, integrating OMICS-based and imaging biomarkers into longitudinal cohorts will be important to define robust and reproducible markers of early MS pathogenesis and to inform the timing of potential interventions before irreversible damage occurs.

### 7.4 Preventive and neuroprotective strategies

Within the framework of a biological continuum, the prospect of preventing MS before clinical onset is increasingly being explored. The near-universal seropositivity for EBV among individuals with MS, together with robust longitudinal evidence linking EBV seroconversion to disease onset, provides a rationale for investigating vaccine-based preventive strategies. A recent review has proposed EBV vaccination as a potential primary prevention approach in carefully selected high-risk populations (for example, EBV-seronegative adolescents with a family history or high genetic risk), and has outlined the scientific and ethical considerations for such trials (160).

Other modifiable risk factors may also offer preventive opportunities. Observational and MR studies support an inverse association between vitamin D levels and MS susceptibility; however, randomized controlled trials in genetically susceptible cohorts are still lacking (161).

In parallel, remyelinating and neuroprotective therapies, though not yet approved for clinical use, are under active investigation (162). Enhancing endogenous repair mechanisms, such as oligodendrocyte differentiation and microglial modulation, remains a major research focus. These strategies may be particularly relevant in preclinical stages, when neuroinflammation and axonal injury are already detectable by imaging and biomarkers, but irreversible damage has not yet occurred (163).

Although preventive and neuroprotective strategies remain largely investigational, they represent promising avenues for future therapeutic development in MS.

### 7.5 Ethical implementation of early diagnosis and prevention

The push toward early identification of MS, possibly during early asymptomatic or radiologically isolated stages, raises important ethical questions. Communicating risk to asymptomatic individuals based on biomarkers or risk scores may provoke psychological distress, changes in self-image, or stigma. Studies in MS highlight the lived burden of receiving a diagnosis: patients often describe shock, uncertainty, and disruption in life planning (164). Additionally, the ethics literature in MS emphasizes that disclosure should be managed not as a one-off event, but as a sustained process attentive to relational needs and uncertainty (165).

A critical dilemma lies in defining the threshold for preventive or neuroprotective interventions. Initiating therapy in individuals who might never convert to clinical MS introduces risks of overtreatment, side effects, and unnecessary burden. The decision must weigh the probability of progression, treatment safety, and patient values. The attitudes of patients and clinicians toward early intervention are not uniform: a recent review found variable preferences and concerns about safety, evidence strength, and timing (166). Moreover, early interventions, especially ones requiring long-term therapy, would demand infrastructure, financial resources, and equitable access. These considerations are magnified when dealing with individuals who are not yet ill but considered “at risk.” The treatment burden perspective is relevant here: patients already facing chronic MS report the workload and side-effects are nontrivial, so applying treatment earlier shifts that burden earlier in life (167).

To responsibly move forward, guidelines should incorporate principles of informed consent tailored to early risk states, shared decision-making, psychological support, and transparent communication of uncertainty. Without such safeguards, earlier diagnosis may confer more harm than benefit, by eliciting unwarranted fear or prompting unnecessary interventions before the natural trajectory of asymptomatic or prodromal phases is fully understood.

### 7.6 Looking ahead

As knowledge from genomics, biomarkers, and advanced neuroimaging continues to expand, clinical pathways in MS may evolve toward earlier and more refined risk assessment strategies. In the coming years, several developments may become feasible, including *targeted risk assessment strategies in selected populations*, such as first-degree relatives or individuals with high polygenic risk, potentially incorporating EBV serostatus or other established risk modifiers. In parallel, *broader use of minimally invasive biomarkers,* such as sNfL and sGFAP, together with advanced imaging techniques, may allow monitoring of subclinical neurodegeneration and neuroinflammation.

A *risk-stratified approach to monitoring and intervention* may emerge, whereby only individuals with the highest estimated risk of conversion, such as those with RIS and spinal cord lesions or CSF evidence of intrathecal immune activation, could be considered for early therapeutic strategies, while others undergo structured surveillance.

*Preventive immunomodulation or neuroprotective interventions* remain investigational but may become feasible as future trials clarify the safety and benefit of intervening before overt clinical disease develops.

Achieving such a shift toward more proactive care will require robust validation studies, clear ethical frameworks, and patient-centered guidelines to ensure responsible implementation.

## 8 Conclusion

Increasing evidence suggests that MS evolves along a biological, clinical and diagnostic continuum, from genetic susceptibility and environmental exposures, through possible prodromal and preclinical stages, to RIS, CIS, and ultimately clinically definite MS. These dimensions should be interpreted as complementary but distinct.

Advanced neuroimaging, cerebrospinal fluid analysis and serum biomarkers such as NfL have contributed to the characterization of the preclinical phase, challenging traditional diagnostic notions and compelling a rethinking of the conventional understanding of disease onset. By the time classical symptoms appear, significant neuroaxonal damage and lesion accumulation may already have occurred. Therefore, recognizing MS as a continuum may support earlier approaches to prevention, diagnosis and treatment.

Recent trials in individuals with RIS showing that DMTs can delay MS conversion support the plausibility of early intervention; however, the prospect of intervening at the biological onset of MS remains an aspirational goal requiring further validation. As diagnostic criteria evolve and the understanding of the MS prodrome expands, clinicians may gain access to tools that support earlier and more refined risk stratification, potentially enabling identification of individuals at higher risk.

This continuum model also has significant implications for future research priorities, namely refining the specificity of prodromal biomarkers, investigating the effectiveness of EBV vaccination and vitamin D supplementation for prevention in high-risk groups, and advancing neuroprotective and remyelinating therapies to preserve CNS integrity from the earliest possible stage.

In conclusion, adopting a continuum model of MS deepens our understanding of disease pathogenesis by integrating its biological, clinical, and diagnostic dimensions, from early susceptibility to overt clinical stages. This framework may also inform more anticipatory approaches to care, including risk stratification and therapeutic decision-making. However, this shift remains largely investigational, and robust prospective evidence is needed to determine whether earlier intervention can meaningfully delay or prevent disability. The challenge ahead lies not only in detecting MS earlier, but in determining when and how to act, guided by careful clinical judgement, ethical considerations, and sufficiently strong evidence.
